# Scanning Electron Microscopy (SEM) Evaluation of the Ultrastructural Effects on Conjunctival Epithelial Cells of a New Multiple-Action Artificial Tear Containing Cross-Linked Hyaluronic Acid, Cationic Liposomes and Trehalose

**DOI:** 10.3390/biomedicines12091945

**Published:** 2024-08-23

**Authors:** Mario Troisi, Salvatore Del Prete, Salvatore Troisi, Daniela Marasco, Michele Rinaldi, Ciro Costagliola

**Affiliations:** 1 Eye Clinic, Department of Neurosciences, Reproductive and Odontostomatological Sciences, Federico II University, 80131 Naples, Italy; ciro.costagliola@unina.it; 2 Service Biotech s.r.l., 80121 Naples, Italy; saldelp@servicebiotech.com (S.D.P.); danielamarasco.servicebiotech@gmail.com (D.M.); 3 Ophthalmologic Unit, Salerno Hospital University, 84100 Salerno, Italy; salvatore.troisi@gmail.com

**Keywords:** trehalose, cross-linked hyaluronic acid, liposomes, conjunctival trophism, artificial tear persistence, eyedrop residence time, scanning electron microscopy (SEM), conjunctival microvilli, dry eye disease (DED), ocular surface disease, dry eye treatment, dry eye diagnosis, tear film

## Abstract

The authors performed an ex vivo and in vivo evaluation of the ultrastructural effects on the conjunctival epithelial cells of a new multiple-action tear substitute containing cross-linked hyaluronic acid, lipids and trehalose (Trimix^®^), using scanning electron microscopy (SEM) with conjunctival impression cytology. The ex vivo study highlights the persistence and distribution of the product at 5 and 60 min on a monolayer of conjunctival epithelial cells and an increase in microvilli density at the 60 min evaluation. In vivo examination was conducted on three subjects with different grades of ocular surface inflammation, treated with one drop of the product twice daily for thirty days. At the baseline (T_0_) and twelve hours after the last administration of the tear drop (T_30_), impression cytology of the upper bulbar conjunctiva for SEM evaluation of conjunctival epithelial cells was carried out. Slit lamp examination (SLE), corneal and conjunctival Fluotest, tear film break-up time (TBUT), and ocular surface disease index (OSDI) questionnaires were also performed to correlate the ultrastructural results with the clinical findings. After 30 days of treatment, a significant improvement in all clinical and symptomatic parameters and in the condition of the ocular surface was detected, with microvillar regeneration and strengthening in all the patients, and a complete restoration in 2/3 of them. The persistence and distribution of the product on the epithelial cells was also noted 12 h after the last administration. The results, therefore, suggest a marked epitheliotropic effect along with a high residence time of the tear substitute.

## 1. Introduction

Dry eye disease (DED) is the most common ocular surface disorder, characterized by insufficient production and/or instability of the tear film. It may affect between 5% and 50% of the population, depending on age, sex and ethnicity [1]. A multitude of tear substitutes are currently available on the market worldwide, with a wide variety of ingredients. Recently, there has been a progressive shift from simple hydrating medications towards complex multi-action combined formulas aimed at disrupting different mechanisms within the vicious cycle of dry eye disease (DED) [2].

The aim of the study is to evaluate with scanning electron microscopy (SEM) the persistence on the ocular surface and the effects on the microvilli of conjunctival epithelial cells of Trimix^®^ eye drops (OFFHEALTH S.p.A, Via Giovanni Paisiello, 10, 50144, Firenze, Italy), a new multiple-action tear substitute based on 0.15% cross-linked hyaluronic acid (HA), 3% trehalose and cationic liposomes comprising stearylamine and phospholipids [3].

Microvilli are protuberances of the plasma membrane which express the vitality of the mucous and epithelial cells; their alteration expresses a condition of cellular suffering in a predictive sense. Therefore, their degree of alteration is significant to establish how much the inflammatory state or toxic conditions affect cellular functionality [4].

Morphological examination of the microvilli therefore allows us to evaluate to what extent the drug being tested is able to carry out a protective action or a healing role at a cellular level by reactivating cellular functions and determining the restoration of the microvilli, or, on the other hand, determine a pathological alteration compared to the starting conditions [5].

The evaluation of the conditions of the microvilli of the epithelial cells was carried out by impression cytology, a noninvasive, easily reproducible technique, and scanning electron microscopy examination [6,7,8,9,10].

The present study is divided into three steps: in vitro, ex vivo, and in vivo phases. The in vitro and ex vivo phases study the drug, its distribution, and its short-term action on conjunctival impressions in healthy subjects. The in vivo phase studies the effects of the product administered bis in die (B.I.D.) for thirty days, taking images of the conjunctival cells of three treated patients (one with mild/moderate grade inflammation, one with severe dry eye, and one healthy subject) twelve hours after the last instillation, to evaluate the action of the product over the long term and if it is able to induce specific changes in the conjunctival epithelium with particular regard to the ultrastructure of the microvillar surface.

## 2. Materials and Methods

### 2.1. Impression Cytology and SEM Examination

Conjunctival epithelium specimens were collected without anesthetic, using the impression cytology method by compressing a fragment of cellulose acetate on the patients’ bulbar upper-temporal conjunctival surface for 3 or 4 s. Specimens were then transferred to a glass slide by compressing the cellulose acetate fragment on the glass slide for 30 s. 

**Figure 1:**
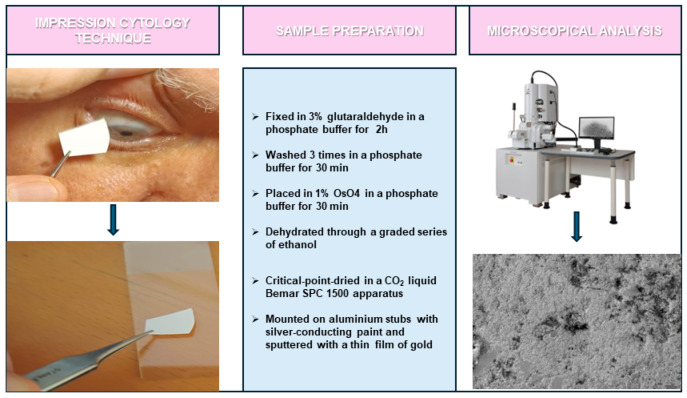
Overview of non-invasive impression cytology for conjunctival sample collection and methodology for sample preparation for SEM analysis.

For SEM, the conjunctival epithelial samples were fixed in 3% glutaraldehyde in a 0.065 M (pH 7.4) phosphate buffer for 2 h at room temperature. Slides were washed three times in 0.065 M phosphate buffer (for 30 min), and then placed in 1% OsO_4_ in 0.064 M (pH 7.4) phosphate buffer for 30 min. Samples were dehydrated through a graded series of ethanol and then critical-point-dried in a CO_2_ liquid Bemar SPC 1500 apparatus (Bomar Co., Tacoma, WA, USA). Specimens were mounted on aluminum stubs with silver-conducting paint, sputtered with a thin (20 nm) film of gold, and observed with SEM Stereoscan 250 (Cambridge Instruments, Cambridge, UK) [6] (Figure 1). 

The microvilli on each sample were first identified at a magnification of 800× in a 1500 μm^2^ field. Then, at a magnification of 8000×, we counted the number of microvilli in each 230 μm^2^ area of the selected field.

### 2.2. In Vitro and Ex Vivo Study Design

In the first phase of the study, a slide was preliminarily prepared with only Trimix^®^ as a sample image identifying the structure of the product to be examined in SEM (CTRL 1).

Furthermore, the presence of the molecule and its interaction with the microvilli were verified, taking two conjunctival samples using the impression method from a healthy subject, without alterations of the ocular surface on slit lamp examination and with OSDI <12. The samples were carried out at 5′ (CTRL 2) and 60′ (CTRL 3) after the administration of 50 microliters of Trimix^®^, fixed with a fixative buffer and processed for visualization in SEM to evaluate the distribution of Trimix^®^ on the conjunctival cell monolayer.

Control groups:

- CTRL1: Drug examined in vitro for structure identification in SEM (in vitro study)
- CTRL2: Conjunctival tissue monolayer evaluated for drug action at 5 min (ex vivo study)
- CTRL3: Conjunctival tissue monolayer evaluated for drug action at 1 h (ex vivo study).

### 2.3. In Vivo Study Design

The study involved the selection of three subjects to be treated with Trimix^®^ for 30 days to evaluate the action of the product on microvillar structures at various degrees of ocular surface inflammation according to the Efron grading scale: one healthy patient (grade 0), one with mild/moderate inflammation (grade 2–3), and the other with marked inflammation (grade 4).

The subjects enrolled in the study were 3 (1 man and 2 women), Caucasian, with an age range between 44 and 55 years (mean 50.33 ± 6.02). Exclusion criteria for this study consisted of the following: (I) topical or systemic therapies in the previous 30 days; (II) allergic conjunctivitis; (III) infections and other ocular surface diseases in the last three months; (IV) previous herpetic keratitis; (V) corneal opacities and ulcers; (VI) pregnancy and breastfeeding; (VII) systemic therapies with steroids, immunomodulators or tetracyclines in the last six months; (VIII) diabetes mellitus; (IX) chronic hepatitis.

At time-0 slit lamp examination (SLE), corneal and conjunctival fluorescein staining according to the Oxford scale (Fluotest), Tear Break-Up Time (TBUT), and cytological sampling of the upper bulbar conjunctiva with the impression procedure with evaluation of microvilli by SEM were carried out. The ocular surface disease index (OSDI) questionnaire was also administered.

Evaluation of the state of the epithelial microvilli was carried out using a specific preset scale (Del Prete et al.) [6], as shown in Table 1.

The same tests were carried out after 30 days of treatment (T_30_) with Trimix^®^ eye drops, administered twice a day. Treatment was stopped in each subject 12 h before sampling. The results after 30 days of treatment with Trimix^®^ are reported.

## 3. Results

### 3.1. In Vitro and Ex Vivo Study

**Figure 2:**
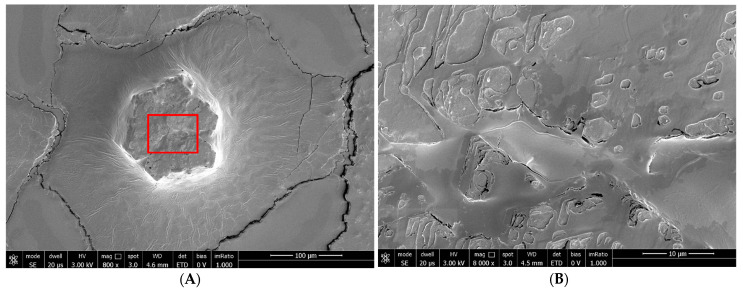
We can see the structure of the tear substitute at SEM at 800× magnification (**A**) and 8000× magnification (**B**) to understand how it is placed on the epithelial surface and interacts with microvilli.
- CTRL2: Evaluation of the action of Trimix^®^ by SEM on a monolayer of conjunctival tissue at 5 min: slight alteration of the microvilli (grade 1); it is possible to observe the persistence of the product on the epithelial surface (Figure 3).

Control group on healthy subject:

- CTRL1: The structure and morphological characteristics of Trimix^®^ and its distribution and interaction with the ocular surface are identified in vitro by SEM evaluation (Figure 2).

**Figure 3:**
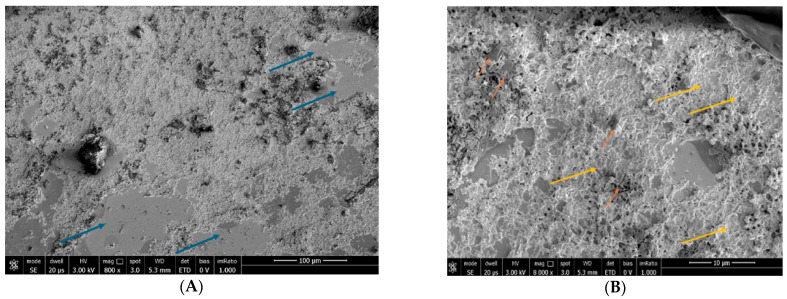
Persistence of Trimix^®^ on conjunctival tissue at 5 min at 800× magnification (**A**) and 8000× magnification (orange arrows) (**B**). Blue arrows indicate areas of cells without microvilli at 800× (**A**). Yellow arrows evidence areas with the presence of microvilli at 8000× (**B**).
- CTRL3: Evaluation of Trimix^®^ action by SEM on a monolayer of conjunctival tissue at 1 h: the product remains and interacts with the cell surface; the entire preparation area is completely covered by microvilli, unlike the microscopic picture detected at five minutes (Figure 4).

**Figure 4:**
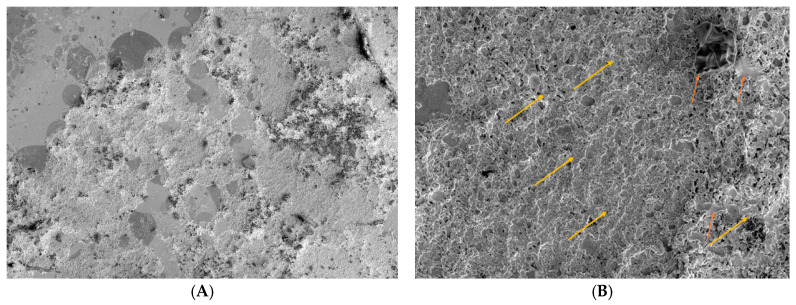
After 1 h, Trimix^®^ persists on conjunctival tissue (orange arrows) and interacts with the cells. The sample evaluations at 800× (**A**) and 8000× (**B**) show an area completely covered by microvilli (yellow arrow), unlike its 5 min counterpart.

### 3.2. In Vivo Study Group

- PATIENT 1: 44-year-old woman with moderate inflammation of the ocular surface (grade 2–3).

**Figure 5:**
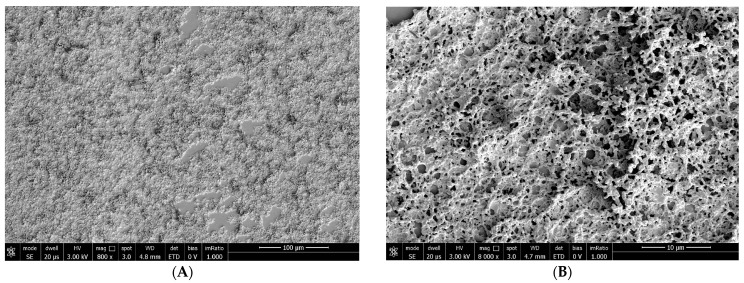
Patient 1 at T0: low alteration of microvilli with not-totally-arborescent shape at 800× (**A**) and 8000× (**B**) magnification.

At T_0_: TBUT: 5 s; corneal conjunctival Fluotest: grade 1; OSDI score: 18; SEM evaluation: low alteration of microvillar distribution and structure (grade 1) (Figure 5).

**Figure 6:**
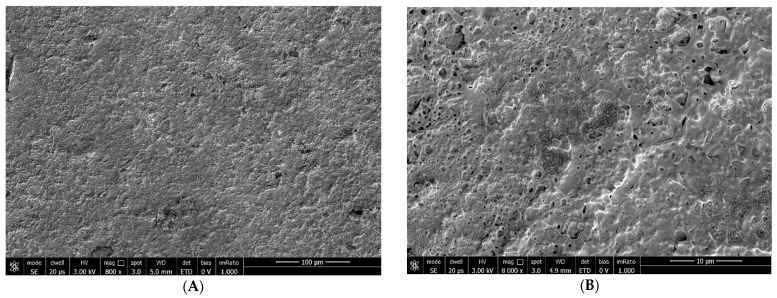
Persistence of the tear substitute 12 h after the last instillation at T_30_ with complete microvillar regularization at 800× (**A**) and 8000× (**B**) magnification.

At T_30_: B.U.T.: 9 s; corneal conjunctival Fluotest: grade 0; OSDI score: 9; SEM evaluation: persistence of the Trimix^®^ on the epithelial surface after 30 days of treatment with complete regularization of the microvillar surface and distribution (grade 0) (Figure 6).

- PATIENT 2: 56-year-old woman with marked inflammation of the ocular surface (grade 4).

**Figure 7:**
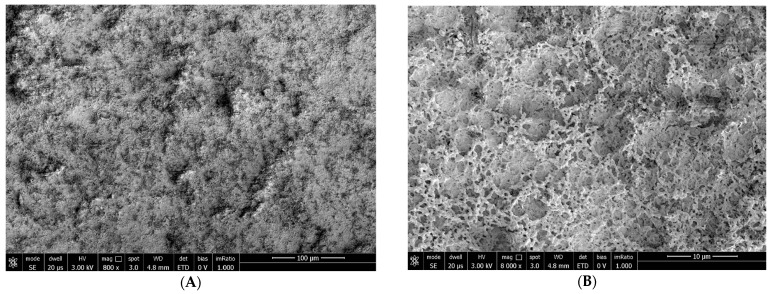
Patient 2 at T_0_: grade 3 alteration of microvilli at 800× (**A**) and 8000× (**B**) magnification.

At T_0_: B.U.T.: 2 s; corneal conjunctival Fluotest: grade 4; OSDI score: 32; SEM evaluation: strong alteration of microvilli (grade 3) (Figure 7).

**Figure 8:**
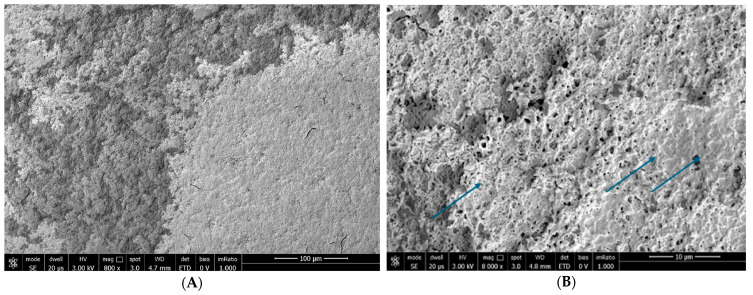
Patient 2 at T_30_: persistence of Trimix^®^ on the ocular surface with microvillar regeneration. In a patient with a previous grade 3 alteration at T0, it is highlighted that the drug covers the cell surface like a blanket, favoring the growth of the microvilli to which it binds strongly (blue arrows). Images at 800× (**A**) and 8000× (**B**) magnification.

At T_30_: B.U.T.: 7 s; corneal conjunctival Fluotest: grade 1; OSDI score: 12; SEM evaluation: Trimix^®^ persistence after 30 days on the epithelial surface; the product penetrated among the microvilli, reinforcing them and restoring the arborescent structure; we can also notice an increased microvillar spread on the conjunctival surface (grade 1) (Figure 8).

- PATIENT 3: 51-year-old man with no signs of ocular surface inflammation (grade 0).

**Figure 9:**
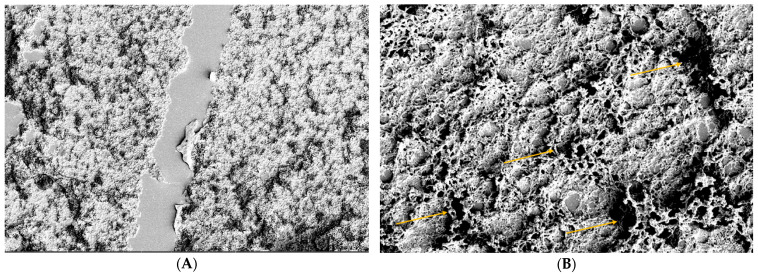
Patient 3 at T_0_: high microvillar distribution at 800× (**A**) and 8000× (**B**) magnification. At 8000× (**B**), we can see how microvilli hold the precorneal tear film, which in the other patients is not visible (yellow arrows). This is an indicator of good condition (grade 0–1).

At T_0_: B.U.T.: 10 s; corneal conjunctival Fluotest: grade 0; OSDI score: 9; SEM evaluation: presence of microvilli with minimum reduction of distribution (grade 0–1) (Figure 9).

**Figure 10:**
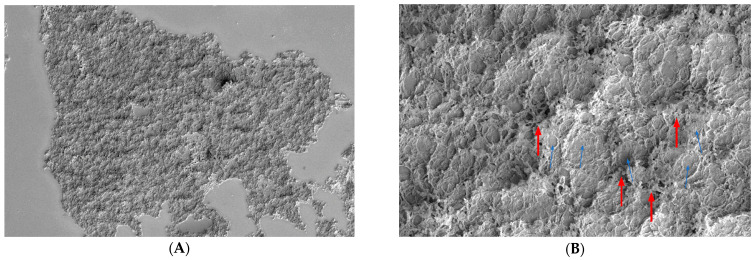
Patient 3 at T_30_: great distribution of the tear substitute on ocular surface at 800× (**A**) and 8000× (**B**) magnification. The density of microvilli grew after the treatment (blue arrow), indicating a complete normalization of the ocular surface, with evidence of Trimix^®^ strongly linked to the microvilli structures (red arrows).

At T_30_: BUT: 13 s; corneal conjunctival Fluotest: grade 0; OSDI score: 6; SEM evaluation: all the surface is covered by the drug; we can see some microvilli that go on through the drug cover (Figure 10).

The results are summarized in Table 2.

After 30 days of treatment, nobody reported adverse reactions.

## 4. Discussion

DED pathogenesis is characterized by a complex loop of cyclic events connecting tear film instability and hyperosmolarity, inflammatory response, and metaplastic changes in ocular surface epithelia. Any therapeutic approach must be targeted at breaking the loop with the aim of preventing the disease’s persistence and progression [11,12,13]. Patients can present different types of DED, with related deficiencies of components of the tear film and abnormalities in the ocular surface epithelium which should be considered in the management of their condition, and more specifically at the time of choosing the most appropriate therapy for each specific case [14]. Tear substitutes, available in different formulations, are usually the first line of treatment for patients with DED. The main types of ingredients used in the composition of tear substitutes are viscosity-enhancing agents, electrolytes, osmo-protectants, oily agents, antioxidants and preservatives [3].

Trimix^®^ is a new-generation multiple-action ophthalmic solution containing cross-linked HA 0.15%, trehalose 3%, liposomes 1% and sterylamine 0.25% [3,15].

Several scientific studies have demonstrated the trophic and protective effects of hyaluronic acid and trehalose on the conjunctival and corneal epithelium [16,17]. It has also been seen that the instillation of eye drops containing the combination of trehalose and sodium hyaluronate produces faster corneal re-epithelialization in the presence of epithelial damage compared to that obtained with eye drops containing sodium hyaluronate alone [2,18,19]. 

One of the main limitations of ocular topical medications is that drops are rapidly eliminated via blinking, baseline and reflex lachrymation, and drainage from ocular surfaces before they can penetrate these tissues in effective quantities. Recent research has focused on developing various drug delivery strategies aimed at improving the topical ocular bioavailability of tear substitutes by providing a prolonged residence time on the cornea and conjunctiva [20,21].

Liposomes, primarily constituting phospholipids, are a good choice of delivery system, owing to their outstanding biocompatibility, feasibility and tenability. They are spherical vesicles with a hydrophilic core and lipidic bilayers. They have been proven to increase the solubility of both hydrophilic and hydrophobic drugs [22,23,24]. The presence of cationic liposomes in Trimix^®^ further contributes to rebalancing the tear film in the lipid component [1,25]. In addition, the positive charge provided by the latter contributes to the generation of electrostatic forces that help in the adsorption of tear film-soluble proteins at the lipid layer interface and, moreover, stabilize this layer. The positive charge of the oil nanodroplets also helps in the homogeneous spreading of the tear substitute on the negatively charged ocular surface [3].

The elevated residence time of this tear drop is also explained by the cross-linked structure of HA. Cross-linking is a chemical strategy that aims to increase the rigidity of the polymer network (i.e., the gel viscoelasticity), extend its permanence at the site of application and decrease its susceptibility to enzymatic degradation, thus reducing the daily number applications of a formulation [26]. 

Analysis of the ocular surface constitutes a crucial aspect of the diagnosis and treatment of dry eye [25,27,28,29]. In our work, the effectiveness of this combination is evaluated for the first time by SEM examination of the microvilli, which represent a very sensitive index of the degree of well-being of the ocular surface, as demonstrated by previous studies [6,30,31,32].

Conjunctival microvilli are microscopic cellular membrane protrusions on apical epithelial cells which increase the surface area available for tear adherence. The deep layer of the tear film binds to the microvilli of the epithelial cells [6]. Furthermore, transmembrane mucins are anchored at the ends of the epithelial surface, thus ensuring its wettability [4]. The microvilli and glycocalyx of the conjunctival epithelium provide support to the tear film, preventing it from leaking from the ocular surface due to gravity [5]. Pathological alterations of microvilli affect the tear film stability and, conversely, disfunctions of tear film composition can lead to a suffering epithelium (dry-eye syndrome) [31].

The present study was carried out by means of cytological impression sampling of the bulbar conjunctiva and SEM examination. Impression cytology was first introduced in 1977 when it was noted that the Millipore VSWP 0.025 μm membrane filter or other absorbent filters remove, in addition to mucous secretions from the conjunctiva surface, even sheets of epithelial cells, including goblet cells, that can be subjected to microscopic examination [32]. Several scientific publications correlate clinical alterations in the ocular surface to the reduction in the microvilli count evaluated by SEM. In particular, Cennamo Gi et al. report a significant reduction in microvillar counts in patients undergoing glaucoma therapy compared to untreated controls [33].

Tsubota’s group, in another study, reports that the mean number of mucosal microvilli was significantly lower in chronic GVHD compared to samples from Sjogren’s syndrome or normal subjects, and the microvilli were significantly shorter, with a lower height-to-width ratio and a significantly lower mean number of secretory vesicles; these are thought to be important factors that influence the stability of the tear film layer and contribute to the severity of GVHD-related dry eye [34]. Microvilli alterations have also been detected in contact lens wearers [35].

On the other hand, studies carried out on healthy subjects [36] or those suffering from dry eye [37], show that tear substitutes can induce specific changes in the conjunctival epithelium, particularly in the ultrastructure of the epithelial cells of the conjunctiva and goblet cells.

The first phase of our study consisted of an ex vivo examination carried out by impression cytological sampling and SEM analysis on a clinically healthy subject, which showed persistence of Trimix^®^ on the cell surface at 5 and 60 min, indicating a high residence time of the product on the ocular surface. The sampling carried out after 5 min highlighted a modest alteration of the microvilli, which was completely restored after 60 min of application of the product, with an increase in the concentration of microvilli. The SEM findings also highlighted a homogeneous stratification of the product on the epithelial surface, presumably favored by the cross-linked structure of the hyaluronic acid, and a strong interaction of the Trimix^®^ with the microvilli, leading to a normalization of the ocular surface.

The in vivo study confirmed the trophic effect of the product, which was even more evident after thirty days of treatment, both in clinically healthy subjects and in patients with moderate or severe alterations of the ocular surface. Furthermore, in each of the subjects examined, the stratification and persistence of Trimix^®^ on the epithelial surface was detected at T_30_, 12 h after the last administration of the product, the structure of which had previously been identified during the in vitro study.

The results obtained suggest a protective and therapeutic effect of this medical product and not just a symptomatic one. In fact, prolonged administration made it possible to obtain a notable increase in the concentration of microvilli and an improvement or complete normalization of the microscopic picture of the ocular surface.

The concordance of the evaluations carried out ex vivo and in vivo corroborates the results obtained, showing strong evidence of the epithelial–trophic and protective effects of Trimix^®^. The clinical data relating to the stability of the tear film, assessed with the BUT test, and the subjective symptoms, measured via the OSDI questionnaire, also show agreement with the morphological data detected by SEM analysis. Finally, the persistence of Trimix^®^ in the conjunctival smear of all the subjects examined 12 h after the last instillation indicates a high residence time of the product on the ocular surface––definitely superior to the studies on various tear substitutes reported in the literature [38,39,40,41,42,43,44,45]. With such a prolonged residence time, there is more time for the drug to act locally on mucosal surfaces and to penetrate deeper ocular tissues to reach its target and exert its therapeutic and regenerative actions.

## 5. Conclusions

The ex vivo therapeutic effects of Trimix^®^, even at one hour from administration, demonstrated a great capacity to protect and regenerate the microvillar surface, which can be associated with the persistence of the product on the ocular surface. All subjects treated in the in vivo phase showed an improvement in the state of the microvilli at T_30_, with complete normalization of the epithelial surface in two of the three treated subjects and a significant increase in microvilli in all cases. Furthermore, the persistence of the product 12 h after its last administration is demonstrated in subjects treated for thirty days by SEM analysis of the conjunctival sampling using the impression method. This is the only study we have found in the literature that demonstrates the persistence of such a prolonged tear substitute, that if confirmed by further studies could suggest its potential for even twice-daily administration.

## Figures and Tables

**Table 1:** Classification of conjunctival microvilli using Del Prete et al.’s scale [6].

Grade 0	Grade 1	Grade 2	Grade 3	Grade 4
Microvilli on site	Microvilli on site	Microvilli on site	Microvilli on site	Smooth area for microvilli absence
Normal surface	Normal surface	Low alteration of the surface	High alteration of the surface	High alteration of the surface
High microvillar distribution	Low microvillar distribution	Microvillar distribution on spot	Microvillar sensible reduction with spotted smooth areas	Microvillar absence
Arborescent structure of microvilli	Structure of microvilli not totally arborescent	Pseudomicrovilli	Pseudomicrovilli	Smooth surface, moon surface

**Table 2:** For each patient, the OSDI score, Fluotest, TBUT and SEM evaluation of the impression cytological sample of the bulbar conjunctiva are reported at T_0_ and T_30_.

Patients	OSDI Score		Fluotest		TBUT		SEM Evaluation	
	T_0_	T_30_	T_0_	T_30_	T_0_	T_30_	T_0_	T_30_
F, 44 years old	18	9	1	0	5″	9″	1	0
F, 56 years old	32	12	4	1	2″	7″	3	1
M, 51 years old	9	6	0	0	10″	13″	0–1	0

## Data Availability

All data are provided in the main text.
